# Computed Tomographic Measures of Funnel-Shaped Lumbar Vertebral Canal and Articular Process Dysplasia Malformations Differ Between German Shepherd and Belgian Malinois Military Working Dogs

**DOI:** 10.3389/fvets.2020.00275

**Published:** 2020-05-27

**Authors:** Constance J. Dragicevich, Jeryl C. Jones, William Bridges, Heather Dunn

**Affiliations:** ^1^Department of Animal and Veterinary Sciences, Clemson University, Clemson, SC, United States; ^2^South Carolina Translational Research Improving Musculoskeletal Heath Center, Clemson, SC, United States; ^3^School of Mathematical and Statistical Sciences, Clemson University, Clemson, SC, United States; ^4^Department of Bioengineering, Clemson University, Clemson, SC, United States

**Keywords:** spine, multi-level stenosis, sports medicine, deep phenotyping, athlete

## Abstract

Researchers who study the selection and breeding program criteria for military working dogs aim to help maximize the years of active duty service. Computed tomographic (CT) quantitative phenotyping has been previously described as a method for supporting these research studies. Funnel-shaped lumbar vertebral foramen malformations have been previously described in Labrador retriever military working dogs and proposed to be risk factors for impaired arterial perfusion of nerve tissues during exercise. Articular process dysplasia malformations have been previously described in varying dog breeds and proposed to be risk factors for articular process degenerative joint disease and vertebral foramen stenosis. Aims of this retrospective, cross-sectional study were to describe quantitative CT phenotyping methods for characterizing funnel-shaped lumbar vertebral foramina and articular process dysplasia malformations and to apply these methods in a comparison between groups of German shepherd and Belgian Malinois military working dogs. A military working dog hospital's database was searched for German shepherd and Belgian Malinois dogs aged <6 years that had CT scans of the lumbosacral region during the period of 2008–2016. Observers unaware of CT findings recorded available clinical data for each of the dogs. An observer unaware of clinical data recorded CT measures of funnel-shaped lumbar vertebral foramina and articular process dysplasia malformations for each of dogs and each of the lumbar vertebrae that were available in the scans. A total of 59 dogs were sampled: 41 German shepherd and 18 Belgian Malinois. Articular process dysplasia and funnel-shaped vertebral foramen phenotypic traits were present in both breeds in this sample, with the frequency and quantitative measure of these traits being greater in German shepherd dogs and heavier dogs. Lower weight dogs had a lesser degree of a funnel-shaped foramen at all sampled vertebral locations. A consistent relationship between articular process dysplasia measures and body weight was not seen. Computed tomography measures of funnel shaped vertebral foramina were greater in German shepherd vs. Belgian Malinois dogs at the L7 vertebra (*P* < 0.01). The CT measures of cranial articular process dysplasia were greater in German shepherd vs. Belgian Malinois dogs at the L4 (*P* < 0.01) and L5 (*P* < 0.05) vertebrae.

## Introduction

Breeding and procurement programs for working dogs aim to select dogs with the most desirable phenotypic traits for mission-specific working tasks and the highest likelihood for maximizing years of active duty service (1–6). Development of quantitative, deep phenotyping methods is important for supporting researchers who study the criteria used by these programs because these methods allow the use of stronger statistical comparison tests (7). Clinical phenotyping can also be insensitive in stoic, high-drive working dogs due to their tendency to mask clinical signs until disease is advanced (2). Degenerative joint disease and cauda equina syndrome have been reported to be important causes of death or euthanasia in military working dogs (1). Lumbosacral disease has been reported to be a predominant cause of euthanasia or retirement in police working dogs (5). German shepherd dogs and Belgian Malinois are widely used as police and military working dogs around the world (3, 5, 8).

“Funnel-shaped” vertebral foramen malformations have been described in Doberman Pinschers and Labrador retrievers (7–10). This type of malformation has been defined as a trapezoidal or cone-shaped vertebral foramen, with the cranial portion of the foramen being smaller than the caudal portion. A previous study in Labrador retriever military working dogs proposed that this malformation is an undesirable phenotypic trait in the lumbosacral region of high-performance canine athletes due to the potential for impaired arterial blood flow in the cauda equina during strenuous exercise (7). Articular process (facet) dysplasia has been defined as absence (aplasia), incomplete formation (hypoplasia), or increased size (hyperplasia) of the cranial or caudal articular processes (11, 12) and has been described in German shepherds and other breeds (9, 12–15). Dysplasia and/or aplasia of the articular processes in the cervical, thoracic, and vertebral spine has been associated with spinal stenosis at corresponding locations in dogs (9, 12, 13, 15). Abnormal shapes or variable joint angles for articular processes have been described as risk factors for biomechanical instability of the vertebral column and degenerative disease (12, 14–16). Computed tomography (CT) has been previously established as a non-invasive, method for quantifying canine lumbosacral vertebral morphology (7, 14, 17).

The objective of the current, preliminary study was to provide background for future research studies by describing quantitative CT phenotyping methods for characterizing funnel shaped lumbar vertebral foramina and articular process dysplasia malformations and to apply these methods in a comparison between groups of German shepherd and Belgian Malinois military working dogs. The research hypothesis was that CT measures for these three malformations would be greater in German shepherd vs. Belgian Malinois breed groups.

## Materials and Methods

This study was a retrospective, cross-sectional design. The sample size was based on convenience sampling, i.e., the number of animals that met inclusion criteria during the records search period. With hospital director approval, an ACVR-certified veterinary radiologist (J.J.) searched the database of a tertiary referral military working dog hospital (LTC Daniel E. Holland Military Working Dog Hospital at Lackland Joint Base, San Antonio; IACUC Exempt Protocol No. 2019-04) and retrieved medical record and CT data for dogs that had CT scans of the lumbosacral region from 2008 to 2016. This range began with the year that the CT machine was installed at the hospital, up through the most recent full calendar year of data at the time of initiation of this study. For inclusion in the study, dogs had to be German Shepherd or Belgian Malinois breed, and aged ≤ 6 years at the date of their first presentation for lumbosacral region CT scanning. Dogs of this age group were chosen based on a previous study reporting evidence that dogs younger than 6 years of age were more likely to successfully recover from treatment for lumbosacral stenosis (2). This age group choice was also intended to help minimize possible effects of degenerative disease on vertebral measurements.

An undergraduate research student assigned a research number to each dog and created a randomized list of dogs based on these numbers (https://www.random.org/). In consultation with the veterinary radiologist, a graduate student (C.D.) recorded CT measurements in this random order without knowledge of medical record findings (Horos for Mac, version 3.0.1, www.horosproject.org; Mac Pro, Apple Inc., Cupertino, CA). Hip-extension CT studies were used for all measurements. Measurements were acquired for each available lumbar vertebra in the lumbosacral region. For purposes of this study, the lumbosacral region was defined as L4-S1 (7). If both bone and standard algorithm studies were available, the bone algorithm studies were selected. If scans were acquired using variable slice thicknesses, scans acquired with the smallest available slice thickness were selected. If more than one scan was acquired for a dog, the first scan was selected. Measurements were made using the software program's three-dimensional (3D) multiplanar reformatting (MPR) tool and standard bone window settings (WW: 1,500, WL: 300). For each slice location, the 3D MPR tool was used to correct for any positioning obliquity before making transverse area measurements.

**Figure 1:**
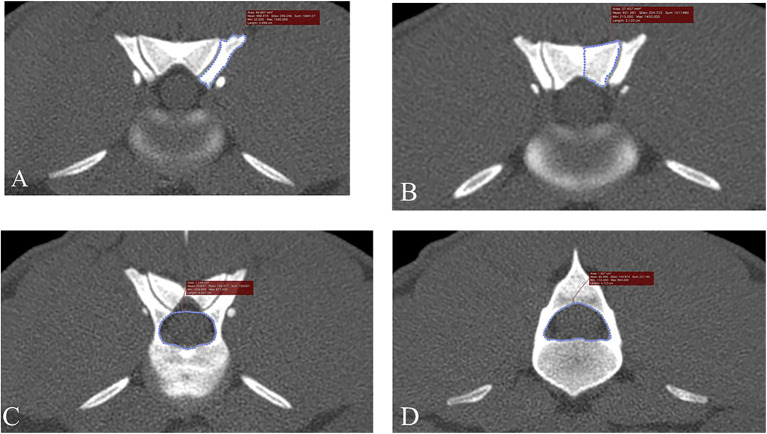
Transverse, bone window CT images illustrating measurement methods for quantifying articular process dysplasia and funnel shaped vertebral foramen phenotypes (Slice thickness 0.625 mm, WW 1500, WL 300, Bone Algorithm). **(A)** Left cranial articular process transverse area; **(B)** left caudal articular process transverse area; **(C)** cranial vertebral foramen transverse area; **(D)** caudal vertebral foramen transverse area. Images are oriented so that dorsal is at the top, ventral is at the bottom, and the patient's left is to the viewer's right.

In addition to randomizing the order of dogs by research number and blinding the observer to medical record findings, bias for CT measurements was further minimized by determining the order for the side of measurements (right, left) using a coin flip. To minimize outside effects of intra-observer variation, each CT measurement was made in triplicate and averaged values were used in further analyses. Figure 1 illustrates how measurement decisions were made. Articular process transverse areas were recorded in mm^2^ and foramen transverse areas were recorded in cm^2^. Articular process transverse area was measured using a modified version of previously described methods (9). The articular process measurement area decisions were made in the same way as with foramen areas described above; a transverse plane was placed bisecting the intervertebral disc space, and the first slice that showed both cranial and caudal articular processes was used for measurements (Figures 1A,B). The medial border of the caudal articular process was defined as a vertical line traced in the median plane, from the dorsal margin of the articular process to the dorsal margin of the vertebral foramen. When osseous proliferations were present, they were included in articular process ROI tracings only if they were of the same opacity as the adjacent bone. Cranial and caudal vertebral foramen transverse area measurements were performed using freehand tracing of regions of interest (ROIs), and were based on previously published methods (7, 17) (Figures 1C,D). The decision for choosing slice locations to perform measurements of the cranial foramen area was made by beginning at a slice bisecting the intervertebral space of the vertebra of interest and the vertebra cranial to it, and then moving caudally one slice at a time until the pedicles around the foramen opening were fully enclosed on transverse view. Likewise, the caudal foramen measurement decision was made by beginning at the intervertebral space between the vertebra of interest and the vertebra caudal to it, and then moving cranially slice-by-slice until the caudal foramen opening was fully enclosed. Data were recorded on a hard-copy data recording sheet first and then transferred to a spreadsheet (Microsoft Excel for Office 365, version 1812).

All statistical analyses were performed by a graduate student (C.D.) in consultation with a statistician (W.B). The following variables were evaluated (see Table 1 for formulas): cranial articular process transverse area difference (CrAPd), cranial articular process transverse area ratio (CrAPp), caudal articular process transverse area difference (CdAPd), caudal articular process transverse area ratio (CdAPp), vertebral foramen transverse area difference (VFD), and vertebral foramen transverse area ratio (VFP). The primary statistical model used in this study included terms for breed, individual dogs within each breed, and vertebral location. Analysis of variance was used to calculate F-tests for the effects of breed and vertebral location. This model was used to make the comparisons among breeds and among locations. Some additional (or secondary) statistical models were used to determine if the covariates of age, sex, and weight class [dogs were assigned to lower (<32.884 kg) and heavier (>32.885 kg) weight classes] had any influence on breed and locations comparisons. These models included the terms from the primary model, one of the covariates, and the interactions of the covariate with breed and location. Analysis of covariance was used to calculate F-tests for the effects of the covariates. All statistical calculations were performed using statistical analysis software (JMP Pro for Windows 10, Version 13.2.0, SAS Institute Inc., Cary, NC) and statistical significance was defined as *P* < 0.05.

**Table 1:** CT measures of vertebral malformations used for breed comparisons.

**Vertebral malformation**	**CT measure**	**Abbreviation**	**Formula^**a**^**
Cranial articular process dysplasia	Cranial articular process transverse area ratio	CrAPp	[(R CrAP-L CrAP)/R CrAP]*100
	Cranial articular process transverse area difference	CrAPd	R CrAP – L CrAP
Caudal articular process dysplasia	Caudal articular process transverse area ratio	CdAPp	[(R CdAP – L CdAP)/R CdAP]*100
	Caudal articular process transverse area difference	CdAPd	R CdAP – L CdAP
Funnel-shaped vertebral foramen	Vertebral foramen transverse area ratio	VFP	[(Caudal foramen transverse area – Cranial foramen transverse area)/Caudal foramen transverse area]*100
	Vertebral foramen transverse area difference	VFD	Caudal foramen transverse area – Cranial foramen transverse area

a *R, right; L, left; Cr, cranial; Cd, caudal, AP, articular process; Ca, foramen*.

## Results

Descriptions of the sampled dogs are provided in Table 2. A total of 59 dogs met initial inclusion criteria, comprising 18 Belgian Malinois and 41 German shepherds. All dogs were scanned based on a consensus opinion between the primary care clinician and a veterinary radiologist. Reasons listed for scanning in the “other” category of Table 2 included the following: tenesmus, hypertension, possible torn ureter, pain on palpation, previous lumbosacral surgery, left metacarpal five fracture, possible pain, and electromyography abnormalities. One dog underwent lumbosacral surgery prior to scanning and a small defect was noted in the margins of the cranial L5 foramen, however this defect did not alter the ability to perform region of interest tracing and the dog was therefore included in analyses. Entire L4 through L7 vertebrae were available in CT scans of 52 dogs. Portions of L4 were not available for five dogs, portions of L4 and L5 were not available for one dog, and portions of L7 were not available for one dog. Missing vertebral locations were listed as “not available” for analyses. The German shepherd group included 32 males and nine females. The Belgian Malinois group included 10 males and eight females. Weights ranged from 20.4 to 43.8 kg. The average weight of the German shepherds was 32.84 ± 4.42 kg and the average weight of the Belgian Malinois was 30.58 ± 5.48 kg.

**Table 2:** Clinical characteristics of sampled dogs.

**Variable**	**Category**	**German shepherd *N* = 41**	**Belgian Malinois *N* = 18**
Average age (years, SD)		3.68 ± 1.78	4 ± 1.94
Age range (years)		1–6	1–6
Average weight (kg, SD)		32.84 ± 4.42	30.58 ± 5.48
Weight range (kg)		24.95–44.0	20.41–41.28
Number of dogs in each weight class^a^	Lower	21	12
	Upper	20	6
Sex	Male	32	10
	Female	9	8
Reasons for CT scanning	Neurologic deficits	15	2
	Lumbosacral pain	15	5
	Vertebral pain (other than lumbosacral)	1	0
	Problems with hindlimbs	22	6
	Inability/reluctance to perform certain actions	3	2
	Possible lumbosacral disease	4	1
	Diagnosed with vertebral disease	6	1
	Unspecified lameness	3	2
	Research study	4	0
	Other	4	3
	Reason not specified	1	1

a *Weight class defined as lower if ≤ 32.884 kg and upper if >32.884 kg. “Other reasons” included dogs that were scanned for reasons unrelated to musculoskeletal or neurological issues of the lumbosacral region or hindlimbs. Twenty dogs had more than one reason for scan listed*.

All animals were scanned in dorsal recumbency, using the same multi-slice CT scanner (Lightspeed VCT, GE Medical Systems, Pewaukee, WI). Technical parameters were as follows: slice thickness 0.625–1.000 mm, kVp 120, mAs 16–700, and matrix 512 × 512 mm. While hip extension scans were available for all included dogs, the degree of hip extension was not standardized. Some dogs were positioned with hips maximally extended and stifle joints adducted (OFA position) and some dogs were positioned with hips in a relaxed extension position and stifle joints abducted (frogleg position).

**Figure 2:**
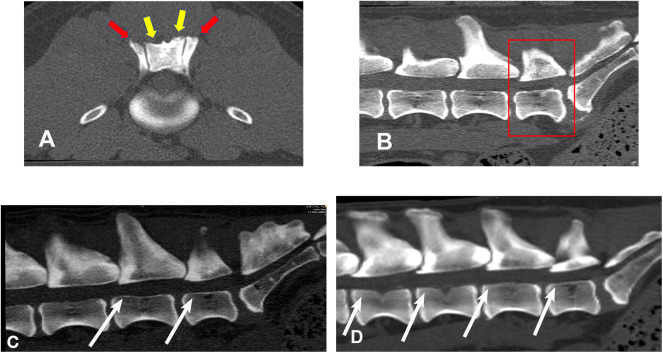
Bone window, CT images illustrating examples of articular process dysplasia and funnel-shaped vertebral foramen malformations (slice thickness 0.625 mm, WW 1500, WL 300, Bone algorithm). **(A)** transverse planar image illustrating articular process dysplasia involving cranial (red arrows) and caudal (yellow arrows) articular processes; **(B)** sagittal planar image illustrating funnel-shaped vertebral foramen at L7 (boxed region); **(C)** sagittal planar image illustrating funnel-shaped vertebral foramina at L6 and L7 (white arrows); **(D)** sagittal, planar image illustrating funnel-shaped vertebral foramina at L4, L5, L6, and L7 (white arrows). Sagittal planar images are oriented so that dorsal is at the top, ventral is at the bottom, cranial is to the viewer's left, and caudal is to the viewer's right.

**Figure 3:**
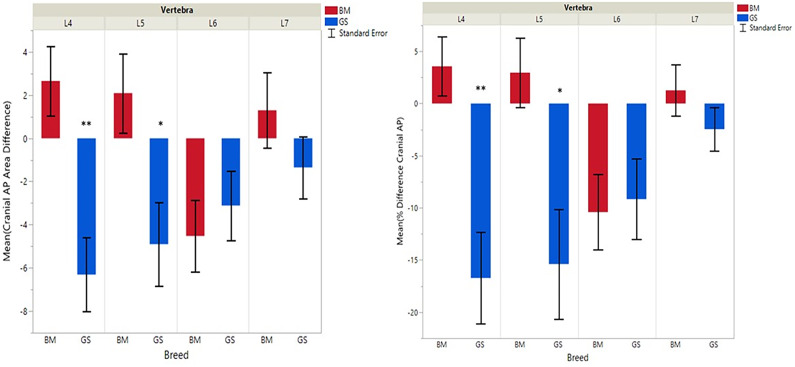
Graph illustrating differences for right vs. left cranial articular processes and mean transverse area ratios (%) (CrAPd and CrAPp). The * indicates a *P*-value of < 0.05, and ** indicates *P* < 0.01.

**Figure 4:**
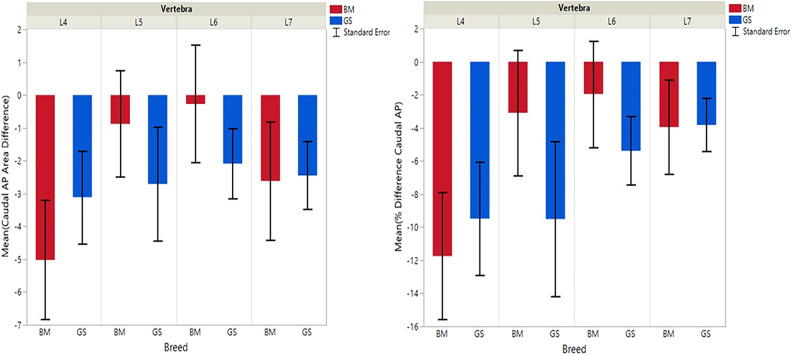
Graph illustrating differences for right vs. left caudal articular processes and mean transverse area ratios (%) (CdAPd and CdAPp ).

**Figure 5:**
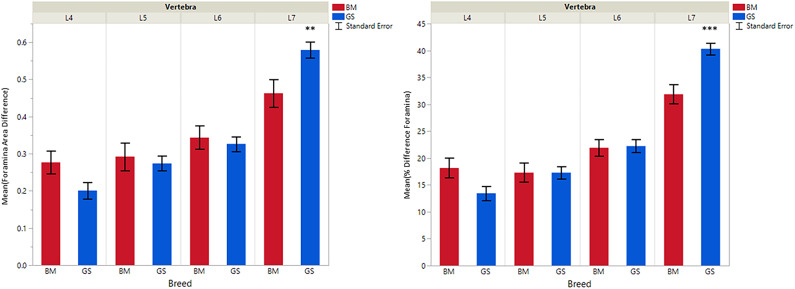
Graph illustrating differences for caudal vs. cranial vertebral foramina and mean transverse area ratios (%) (VFD and VFP). The ** indicates a *P*-value of < 0.01, and *** indicates *P* < 0.0001.

Figure 2 illustrates an example of articular process dysplasia (Figure 2A) and funnel-shaped vertebral foramen malformations (Figures 2B–D). Detailed descriptions of vertebral measurements that were used for calculating the six variables used for breed comparison analyses are provided in Table 3. Figures 3–5 provide graphical breed group comparisons for the six measured variables.

**Table 3:** Vertebral foramen and articular process CT measurements, by breed and vertebra^a^.

**CT measurement**	**L4**		**L5**		**L6**		**L7**	
	**GSD**	**BM**	**GSD**	**BM**	**GSD**	**BM**	**GSD**	**BM**
**R CrAP**	*N* = 37	*N* = 16	*N* = 40	*N* = 18	*N* = 41	*N* = 18	*N* = 41	*N* = 18
**Mean (mm**^**2**^**)**	53.7 (16.9–98.3)	61.0 (30.3–94.3)	47.7 (18.2–86.00)	53.9 (36.6–79.8)	49.6 (17.0–83.3)	51.6 (33.3–72.7)	72.2 (26.2–110.7)	69.6 (44.9–86.0)
**Range (mm**^**2**^**)**								
**L CrAP**	*N* = 37	*N* = 16	*N* = 40	*N* = 18	*N* = 41	*N* = 18	*N* = 41	*N* = 18
**Mean (mm**^**2**^**)**	60.0 (18.9–99.4)	58.4 (33.0–84.0)	52.6 (14.1–90.8)	51.8 (33.3–67.8)	52.8 (14.1–86.5)	56.1 (36.7–73.4)	73.6 (26.4–113.7)	68.3 (50.8–88.3)
**Range (mm**^**2**^**)**								
**R CdAP**	*N* = 39	*N* = 18	*N* = 41	*N* = 18	*N* = 41	*N* = 18	*N* = 40	*N* = 18
**Mean (mm**^**2**^**)**	48.5 (27.9–75.0)	50.3 (33.8–69.7)	44.1 (20.6–70.0)	49.2 (29.0–71.2)	54.8 (27.4–96.1)	56.8 (31.4–76.9)	76.3 (42.3–113.8)	67.2 (47.2–84.4)
**Range (mm**^**2**^**)**								
**L CdAP**	*N* = 39	*N* = 18	*N* = 41	*N* = 18	*N* = 41	*N* = 18	*N* = 40	*N* = 18
**Mean (mm**^**2**^**)**	51.6 (27.8–72.2)	55.3 (32.7–72.1)	46.8 (19.2–74.9)	50.1 (30.4–68.6)	56.9 (26.4–84.4)	57.1 (33.5–72.6)	78.7 (40.1–111.3)	69.8 (35.3–86.56)
**Range (mm**^**2**^**)**								
**CrVF**	*N* = 37	*N* = 16	*N* = 40	*N* = 18	*N* = 41	*N* = 18	*N* = 41	*N* = 18
**Mean (cm**^**2**^**)**	1.21 (0.88–1.56)	1.23 (1.02–1.58)	1.27 (0.96–1.61)	1.33 (1.15–1.67)	1.12 (0.78–1.49)	1.19 (0.98–1.52)	0.85 (0.62–1.16)	0.97 (0.76–1.15)
**Range (cm**^**2**^**)**								
**CdVF**	*N* = 39	*N* = 18	*N* = 41	*N* = 18	*N* = 41	*N* = 18	*N* = 40	*N* = 18
**Mean (cm**^**2**^**)**	1.42 (0.93–1.84)	1.50 (1.23–1.85)	1.52 (1.11–1.95)	1.62 (1.26–2.03)	1.45 (1.01–1.91)	1.53 (1.16–1.93)	1.43 (1.19–1.81)	1.43 (1.09–1.88)
**Range (cm**^**2**^**)**								

*CT, computed tomographic; L4, 4th lumbar vertebra; L5, 5th lumbar vertebra; L6, 6th lumbar vertebra; L7, 7th lumbar vertebra; GSD, German Shepherd dog; BM, Belgian Malinois dog; R, right; L, left; Cr, cranial; Cd, caudal; AP, articular process; VF, vertebral foramen; mm^2^, millimeters squared; cm^2^, centimeters squared*.

German shepherds displayed a wider variance than Belgian Malinois among all six variables at all vertebral locations, except for the cranial vertebral foramen at L7. Analysis of covariance revealed no significant interaction between breed and sex or age among any of the variables at any of the vertebral locations (*P* > 0.05). Breed had a significant effect on CrAP and VFD comparisons. The means of CrAPd and CrAPp at L4 (*P* < 0.005) and L5 (*P* < 0.05), VFD at L7 (*P* < 0.01), and VFP at L7 (*P* < 0.0001) varied between breed groups. When breed was the only factor, there was no significant difference in means between German shepherds and Belgian Malinois for either CT measure of caudal articular process dysplasia (CdAPd and CdAPp) at any of the vertebral locations (*P* > 0.05). The CrAPd and CrAPp means were greater for German shepherds than Belgian Malinois at L4 (*P* < 0.01) and L5 (*P* < 0.05), and there was no significant difference in these measures between breeds at L6 and L7. The VFP and VFD means were greater in German shepherds than Belgian Malinois at L7 (*P* < 0.0001 and *P* < 0.01, respectively).

Breed was significantly affected by the covariate weight for CrAPd at L7 (*P* < 0.05), CrAPp at L5 and L7 (*P* < 0.05), VFP at L7 (*P* < 0.05), and for CdAPd and CdAPp at L4 and L7 (*P* < 0.05). The mean CrAPd and CrAPp values were subjectively greater in German shepherds at L4 and L5 in the lower weight class, and at L4 in the upper weight class. The mean CrAPd and CrAPp values were subjectively greater in Belgian Malinois at L6 in the lower weight class and at L5 and L6 in the upper weight class. Mean CdAPd values were subjectively larger in German shepherds at all vertebral locations in the lower weight class, and were subjectively larger at L4, L6, and L7 for Belgian Malinois in the upper weight class. As a function of weight, German shepherds had subjectively greater mean VFP and VFD values at L7 vs. Belgian Malinois. However, none of these subjective differences were statistically significant.

## Discussion

Intentions of this preliminary study were to provide background for use in future research studies evaluating the selection criteria for military working dogs. Findings supported the study hypothesis in that CT measures of articular process dysplasia and funnel-shaped vertebral malformation phenotypes were greater in German shepherd vs. Belgian Malinois breed groups. However, study findings also indicated that these malformations were present in both breeds. In examining the variable of breed alone, German shepherds had significantly greater right-left articular process dysplasia at L4 and L5, and a significantly more frequent occurrence of funnel-shaped vertebral foramina at L7 than Belgian Malinois. Subjectively, as German shepherds got heavier, the degree of cranial and caudal articular process dysplasia worsened. Among Belgian Malinois, as weight increased, the degree of cranial and caudal articular process dysplasia improved minutely.

For both breeds, mean VFP and VFD values subjectively increased at each individual vertebral location, with the highest subjective differences at L7 (excepting the VFP value for Belgian Malinois at L4). In every dog measured, the cranial vertebral foramen area of L7 was smaller than the caudal foramen area, and for all but three dogs some degree of a funnel-shaped vertebral foramen malformation was also identified at L4, L5, and L6 locations. Based on our review of the literature, this vertebral malformation has not been previously reported in German shepherd or Belgian Malinois dogs. A funnel-shaped vertebral foramen is considered to be a malformation because standard anatomic reference texts describe vertebral foramina as having cranial portions nearly equal in size to caudal portions (18). Primary stenosis has been previously defined in dogs and humans as an abnormally narrow vertebral foramen caused by a congenital or developmental error in vertebral bone formation (19, 20). This primary stenosis can reduce the functional reserve capacity of the vertebral foramen and exacerbate effects of subsequent acquired stenosis due to degenerative disease. Authors of the current study propose that a funnel-shaped vertebral foramen is a form of primary stenosis in dogs. Experimental studies in pigs and dogs have demonstrated evidence that even mild cauda equina compression at more than one level results in impaired venous outflow, increased interstitial pressure in cauda equina nerve tissues located between the levels of compression, and reduced arterial perfusion of these cauda equina nerve tissues (21–23). Human studies have described an increase in these effects during exercise due to higher oxygen demand by nerve tissues and increased congestion of vertebral venous plexus vessels (24).

Dysplasia of the cranial articular processes has been considered uncommon, while caudal articular process dysplasia has been well-documented (11, 12, 15, 25). Current study findings therefore differed from previous studies in that cranial articular process dysplasia was found to be present in both German Shepherd and Belgian Malinois breeds. We did not identify a significant effect of breed alone on CrAPd comparisons, contrary to existing publications describing caudal articular process dysplasia (11, 12, 15). In both breeds and at all locations sampled in the current study, the average left caudal articular process transverse area values were larger than the right. The reason for this finding remains unknown. Overall et al. proposed that repetitive, obsessive-compulsive behaviors, such as spinning and tail-chasing could be exacerbated by excessive confinement (26). Vertebral structures are formed by endochondral ossification during skeletal growth and this process is affected by both internal and external forces (18, 27). It is therefore possible that spinning behaviors in kenneled, military working dogs could be a risk factor for articular process malformations affecting one side more than the other.

The clinical relevance of asymmetrical articular process morphology has been described in several previous publications (2, 13–16). A case report described a young German shepherd with neurological symptoms caused by dysplastic lumbar articular process joints (15). Another study described a common finding of straight-edged articular process joints among German shepherds, whereas other breeds featured rounded articular processes which were more desirable for vertebral stability (14). Three studies associated articular process dysplasia with lumbosacral stenosis and reduction in spine strength and range of movement (13, 15, 16). Three studies have associated lumbar pain with enlarged (hypertrophied) articular processes of the caudal lumbar spine in both dogs and humans (2, 15).

Findings from the current study indicated that weight had a significant effect on breed comparisons for at least one location for all variables except VFD. This finding supported other studies reporting evidence that heavier dogs were at a higher risk for lumbosacral stenosis (7, 8, 28). A surprising finding in this study was a lack of evidence of sex effects on comparisons. This finding did not support previous reports describing evidence that male dogs were at greater risk for lumbosacral lesions (8, 28–30). It is possible that these discordant results occurred because females were lower weight than males on average in our sample. It is also possible that a lack of significance for sex as a covariate in this study could have been caused by unequal group sizes. There was also no significant effect of age in the current study. This is likely due to sample bias in that we chose to include only dogs aged ≤ 6 years. This choice was based on our intention to focus on dogs that were in the age group previously reported to be more likely to recover following treatment for lumbosacral stenosis (2) and also our intention to prioritize early detection.

Findings from the current study also indicated that funnel-shaped vertebral foramen and articular process dysplasia malformations were present in both breeds at L4. It is currently not known whether lumbar vertebrae cranial to L4 may also have exhibited these malformations. One study reported high prevalence of articular process dysplasia in the entire spine in dogs that were euthanized for reasons unrelated to the study (31). Though the author concluded that this high prevalence discounted the clinical significance of these lesions, more recent studies have linked articular process dysplasia to clinical signs of spinal disease in dogs and horses (10, 11, 15, 16, 32). Multi-level bony foramen stenosis has also been reported as a cause of clinical signs of spinal disease in dogs (7, 10, 30, 33, 34). One study identified a moderate to high heritability of several lumbosacral disease-related anatomical features in the lumbar spine in a large sample of German shepherds (34). Another study identified potential candidate genes for lumbosacral stenosis in a sample of 8 military working Labrador retrievers, and recommended that this candidate gene be further evaluated in larger samples and other breeds (35). Repeating the present study with a larger sample size of dogs would allow a “normal” vs. “affected” population to be established. This would be helpful background information to further explore related candidate genes and to more definitively assess the clinical relevance of the phenotypes described in our study.

One of the limitations for the current study was the fact that dog positioning was not standardized. Some dogs were positioned with maximal extension (OFA position) and other dogs were positioned with a relaxed, frog leg extension position at the discretion of the presiding veterinarian. Because of these variations in patient positioning, we chose to focus on measurements of lumbosacral spinal components that were least likely to be affected by positioning, i.e., the vertebral foramina and articular processes. We also used MPR to correct for positioning obliquity to help ensure that true transverse planar slices were used for all measurements. Slice thicknesses for all dogs were either 0.625 or 1.000 mm, therefore effects of slice thickness variation were considered to be negligible. Reader bias was minimized by having one observer who was unaware of clinical findings perform all measurements, by selecting dogs in random order based on research numbers, and by randomizing measurement order for each dog with a coin flip. Intra-observer variability was also minimized by averaging triplicate measurements for all statistical comparisons. Interobserver repeatability was not assessed because one observer made all measurements. Group sizes for sampled dogs were also unequal. The statistician therefore selected analyses that minimized effects of unequal group sizes for this study.

In conclusion, findings from this preliminary study indicated that articular process dysplasia and funnel-shaped vertebral foramen phenotypic traits were present in German Shepherd and Belgian Malinois military working dogs, with the frequency and quantitative measures of these traits being greater in German Shepherd dogs and heavier dogs. Lower weight dogs had a lesser degree of the funnel-shaped foramen phenotype at all sampled vertebral locations. A consistent relationship between articular process dysplasia measures and body weight was not seen. Quantitative CT phenotyping characteristics of these two vertebral malformations may be helpful background for future research studies evaluating the criteria for selection and breeding programs in military working dogs and other high-performance canine athletes. Future longitudinal studies are needed to test associations between these CT phenotypic measures and later development of clinical disease.

## Data Availability Statement

The datasets for this article are not publicly available because of patient confidentiality requirements detailed in the study's IACUC protocol. Requests to access the datasets should be directed to Army Public Health Center ATTN: MCHB-IP-V 8252 Blackhawk Rd. Aberdeen Proving Ground, MD 21010-5403; email usarmy.apg.medcom-aphc.mbx.iph-vet@mail.mil.

## Ethics Statement

The animal study was reviewed and approved by the LTC Daniel E Holland Military Working Dog Hospital at Lackland Joint Base, San Antonio; IACUC Exempt Protocol No. 2019-04.

## Author Contributions

CD, JJ, WB, and HD: conception and design, revising article for intellectual content, and final approval of the completed article. CD and JJ: acquisition of data and drafting the article. CD, JJ, and WB: analysis and interpretation of data.

## Conflict of Interest

The authors declare that the research was conducted in the absence of any commercial or financial relationships that could be construed as a potential conflict of interest.
